# Genome-Wide Identification and Analysis of the Cytochrome B5 Protein Family in Chinese Cabbage (*Brassica rapa* L. ssp. *Pekinensis*)

**DOI:** 10.1155/2019/2102317

**Published:** 2019-12-02

**Authors:** Han Zheng, Xin Li, Lin Shi, Ying Jing, Qingqing Song, Yanan Chen, Lilong He, Fengde Wang, Jianwei Gao, Yuping Bi

**Affiliations:** ^1^College of Life Science, Shandong Normal University, Jinan 250100, China; ^2^Shandong Branch of National Vegetable Improvement Center, Institute of Vegetables and Flowers, Shandong Academy of Agricultural Science, Jinan 250100, China; ^3^College of Life Science, Shandong University, Qingdao 266200, China

## Abstract

Cytochrome B5 (CB5) family proteins play an important role in various oxidation/reduction reactions in cells as the electron donor and are involved in a variety of biotic and abiotic stress processes. However, the function of the *CB5s* in *Brassica rapa* is still unclear. In this study, we carried out genome-wide identification, characterization, and expression analysis of *BrCB5s* in different tissues under adversities and stresses. It was identified that fifteen *BrCB5s* were distributed on different chromosomes, which were classified into seven groups (A-G) according to its phylogenetic relationship. Phylogenetic analysis of the CB5 protein sequences from six species showed that the BrCB5s conduct a close evolutionary process with the CB5s of *Arabidopsis thaliana* and far from those of *Oryza sativa*. Protein interaction analysis showed that 40 interaction patterns were predicted including two Sucrose Transporter 4 subfamily proteins (SUT 4) and Fatty Acid Hydroxylase 2 protein (FAH 2) can interact with most members of BrCB5s. The expression profile analysis indicated that *BrCB5s* were differentially expressed in different tissues, and the transcript abundances were significantly different under various abiotic stresses and plant hormone treatments. Our study provides a basis for a better understanding of the characteristics and biological functions of the CB5 family genes in Chinese cabbage during plant development, especially in plant responses to multiple stresses.

## 1. Introduction

Cytochrome P450 (P450) belongs to a family of heme-binding proteins, which catalyzes multiple functional monooxygenase reactions involved in oxidative metabolism. In plants, *P450s* play a role in the generation of secondary metabolites [1, 2], some of which are synthesized to organize and integrate vital biological processes, and the others are accumulated as defense responders to biotic or abiotic stresses. P450 proteins are important to plants in processes from biosynthesis and metabolism to growth regulation.

Cyt b5 proteins (CB5s), which enhance the turnover of related catabolic enzymes, are important family members of P450 [3]. CB5, anchored to the endoplasmic reticulum (ER), was firstly observed in the larvae of the silkworm *Platysumia cecropia* by Sanborn and Williams in 1950 [4]. CB5s are small (~15kD) heme-binging proteins ubiquitously expressed in animals, plants, fungi, and purple photosynthetic bacteria [5] and function as electron transporters.

Due to their roles in cell detoxification and drug metabolism, CB5s have been studied extensively in animal [6–9]. However, the functions of these proteins have yet to be understood in plants. With the development of molecular biology and sequencing technology, the whole genomes of many species have been known. Multiple CB5 isoforms have been discovered in higher plants. For example, seven CB5s have been found in the model plant *Arabidopsis thaliana* and seventeen in the plant *Oryza sativa*; in contrast, only a signal copy of *CB5* has been discovered in mammals [10]. A hypothesis has been proposed that a large number of CB5 isoforms are needed in response to the increasing number of P450 proteins, as CB5s enhance the activities of P450 proteins by supplying electrons or by physical interaction independent of electron donation [11, 12]. A classical experiment has been designed to understand the relationship between CB5s and P450s via observing the discoloration of flower petals in a *CB5* knockdown mutant of petunia [13]. The result showed that the change of the flower color is accompanied by the decrease in the activity of a biosynthetic P450 enzyme 3‚5′-hydroxylase. Recently, the glucosinolate (GLS) levels of two T-DNA insertion mutants of CB5 isoform C (CB5C) from *Arabidopsis thaliana* were characterized. The first one (*cb5c-1*) was a knockdown mutant with an insertion in the coding region, while the other one (*cb5c-2*) was a “*knockabout*” mutant with an insertion in the 3′ untranslated region of the gene [14]. GLS relates to plant defense [15, 16], and P450 enzymes carry out central catalytic steps in the GLS biosynthetic pathway [17]. These two mutants lead to a subtle and distinct decrease in the levels of GLS under methyl jasmonate treatments. These findings suggest CB5 is required by P450 to be fully functional.

In this study, 12 putative BrCB5s with 3 BrCB5 NADPH-dependent reductases of Chinese cabbage were selected from the Brassica database (http://brassicadb.org/brad/) [18]. We performed a genome-wide bioinformatics analysis of the BrCB5s, including genome location, gene structure, and evolutionary divergence. We identified the expression patterns of these BrCB5s by quantitative real-time PCR (qRT-PCR) in different tissues and in response to various treatments. In addition, we conducted further experiments on the functional characterization of the BrCB5s in Chinese cabbage.

## 2. Results

### 2.1. Identification and Chromosome Location of Chinese Cabbage CB5 Gene Family Members

There were 7 CB5s in *Arabidopsis*, named *At1G26340*, *At1G60660*, *At2G32720*, *At2G46650*, *At5G17770*, *At5G48810*, and *At5G53560* (https://www.arabidopsis.org). For the homologous proteins, fifteen *BrCB5s* were identified from the Brassica database by blasting on the site http://brassicadb.org/brad/ (Table.  1). The *BrCB5s* (a to o) were assigned according to their distribution on chromosomes (Table 2, Supplementary file 1. [Supplementary-material supplementary-material-1]). Chromosomes 02, 04, and 05 have two *BrCB5* genes and the chromosomes 03 and 09 have three, and the last three chromosomes have only one gene.

The data were downloaded from the Brassica database (http://brassicadb.org/brad/). ^a^tPCK Chr: chromosome of translocation the Proto-Calepineae Karyotype, the ancestral karyotype of the Brassicaceae family. ^b^LF: less fractioned subgenome. ^c^MF1 and MF2: more fractioned subgenomes.

### 2.2. Gene Structures and Conserved Motifs of *BrCB5s*

**Figure 1:**
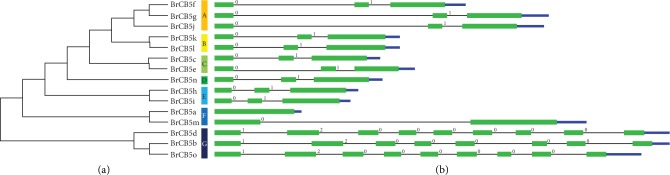
Chinese cabbage *BrCB5* gene family. (a) Phylogenetic relationships among the translated BrCB5 proteins. A-G means the subfamilies of BrCB5s were divided according to their phylogenetic relationships. (b) Intron/exon structure of the *BrCB5* genes.

Intron/exon regions of the *BrCB5s* were identified by aligning the CDSs to the genomic sequence. The results showed that all the *BrCB5* gene sequences contained introns except *BrCB5a.* Although the number of introns varied from zero to eight, most of the genes (10 out of 15) contained two introns. Two genes contained eight introns, and three genes contained seven, one, and zero introns, respectively (Figure 1(b)). Additionally, the number of introns in the genes of the same subfamily of *BrCB5s* was not always the same. For instance, *BrCB5a* and *BrCB5m*, which belong to the same subfamily F, contained zero and one introns, respectively (Figure 1).

Simple sequence repeat (SSR) markers are extensively used in plant genetic mapping and molecular breeding due to genetic codominance abundance, wide distribution in genomes, multiallelic variation, high reproducibility, and high level of polymorphisms [19]. In this study, 17 SSR markers, including eleven di-, five tri-, and one tetranucleotide motifs, were detected in the 15 BrCB5s using the online SSR identification tool SSRIT (Table 3). BrCB5b, BrCB5e, BrCB5j, BrCB5k, and BrCB5n had one SSR marker. BrCB5g, BrCB5l, and BrCB5o had two SSR markers. BrCB5d and BrCB5m had three SSR markers, while BrCB5a, BrCB5c, BrCB5f, BrCB5h, and BrCB5i had no SSR markers. Among these SSR markers, ten were found in introns and seven were found in exons.

To better understand the function of BrCB5s, we used the MEME web server (http://meme.nbcr.net/meme/cgi-bin/meme.cgi) to analyze the domain distribution in BrCB5s (Supplementary file 2. [Supplementary-material supplementary-material-1]). Motif 1, specified as the N-terminal hydrophilic haem-binding domain, was found in 12 of the 15 BrCB5s. The other three CB5s without this motif were found to belong to the NADH-dependent reductase subfamily (Figure 1(a)). Motif 3, specified as the C-terminal hydrophobic region, was predicted to be present in 8 of the 15 BrCB5s, which is less conserved compared with the haem-binding domain, as it is related to the transmembrane function [20]. By MEME analysis, a motif, namely motif 2, was found in 10 of 15 BrCB5s, including BrCB5c, BrCB5 e-l, and BrCB5n.

Multiple sequence alignment was also conducted on the BrCB5 proteins (Supplementary file 3. [Supplementary-material supplementary-material-1]). The result confirmed that all the BrCB5 proteins had a “HPGG” haem-binding domain except these three NADH-dependent reductases (BrCB5b, BrCB5d, and BrCB5o). The BrCB5 family was found to contain a nonconserved C-terminal binding domain. All the results were similar to that by the MEME analysis (Supplementary file 2. [Supplementary-material supplementary-material-1]).

### 2.3. Phylogenetic Analysis and Duplication Events of *BrCB5* Genes

**Figure 2:**
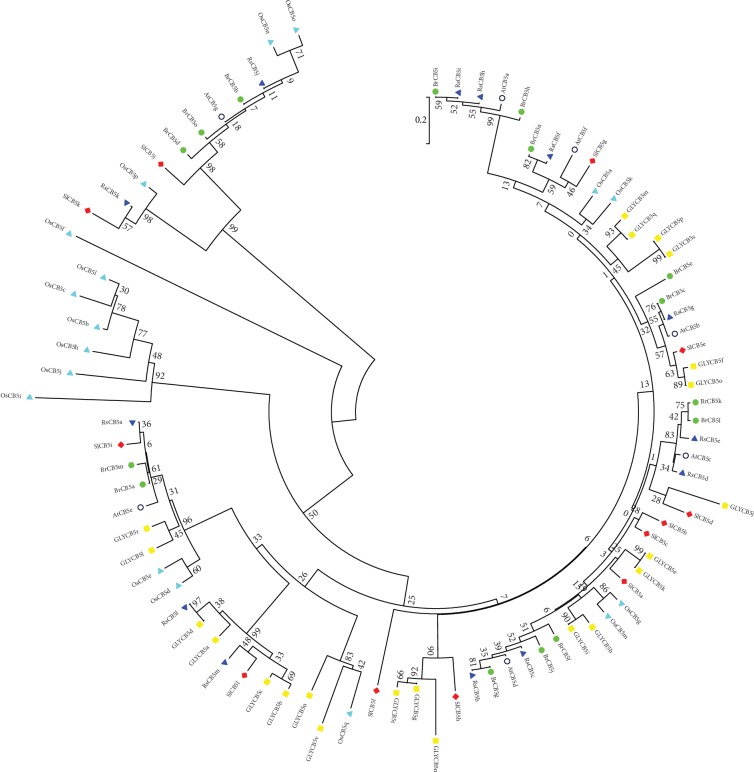
Phylogenetic analysis of CB5 proteins in *Arabidopsis thaliana*, *Brassica rapa*, *Raphanus sativus*, *Oryza sativa*, *Solanum lycopersicum*, and *Glycine max*, whose members were denoted by hollow circles, green circles, blue inverted triangles, cyan triangles, red diamonds, and yellow squares, respectively. The tree, based on the core CB5 domains in the six species, was constructed using the neighbor-joining (NJ) method (bootstrap 1000 replicates) by MEGA5 software.

Classifying genes and phylogenetic analysis are imperative for identifying the functions of a gene family. As Chinese cabbage is one of the most important leafy head vegetables, we analyze its phylogenetic relationship with several model crops: *Raphanus sativus*, *Oryza sativa*, *Solanum lycopersicum*, and *Glycine max*. CB5s involved in these species were obtained using BLAST online tool on NCBI (https://www.ncbi.nlm.nih.gov/). MEGA5 with the bootstrap neighbor-joining method was used to construct the phylogenetic tree (Figure 2), including 15 predicted BrCB5s, 7 *Arabidopsis* CB5s (AtCB5), 13 *Raphanus sativus* CB5s (RsCB5s), 17 *Oryza sativa* CB5s (OsCB5), 12 *Solanum lycopersicum* CB5s (SlCB5s), and 22 *Glycine max* CB5s (GlyCB5s) (including their NADPH-dependent reductases). The result showed that, as a member of the Cruciferae family, CB5s of Chinese cabbage is closely related to *Raphanus sativus* and *Arabidopsis*, which clustered together with Chinese cabbage. But for *Oryza sativa*, lots of its protein members branched alone, indicating the furthest relationship (Figure 2). BrCB5s do also share a far distance with SlCB5s and GlyCB5s, respectively. For the similarity of CB5s in Chinese cabbage and *Arabidopsis*, the most homologous CB5s were identified between BrCB5g/AtCB5d (identity 94.78%), BrCB5c/AtCB5b (identity 92.54%), BrCB5n/AtCB5f (identity 86.67%), BrCB5a/AtCB5e (identity 82.79%), and BrCB5b/AtCB5g (identity 81.49%) (Supplementary file 2. [Supplementary-material supplementary-material-1]).

The syntenic relationship of CB5s between *Arabidopsis thaliana* and *Brassica rapa* is shown in Table 1 with the data downloaded from the Brassica database (http://brassicadb.org/brad/); at the same time, as the great value to the decryption of the evolutionary mechanism, we also analyzed the genome duplication events in the evolutionary process of Chinese cabbage. The result indicated that 15 *BrCB5s* were derived from 7 blocks of 4 tPCK (translocation Proto-Calepineae Karyotype) chromosomes. Coincidently, they were evenly distributed on three subgenomes (LF, MF1, and MF2). There were one to three copies of *BrCB5s* syntenically corresponding to an *AtCB5*. For example, *BrCB5j*, *BrCB5g*, and *BrCB5f* corresponded to *AtCB5d* in the J block; *BrCB5o*, *d*, and *b* corresponded to *AtCB5g* in the R block, while the other *BrCB5s* were either duplicated or singletons.

### 2.4. Protein Interaction Analysis of BrCB5s

The knowledge of the functional interactions of proteins is indispensable to a widely understanding of the molecular machinery. The STRING database (http://string-db.org/) is known as an online tool on which we can get predicted protein-protein association information related to our target proteins. In this study, 40 predicted interactive candidates of BrCB5 proteins were identified (Table 4).

Among these candidates, two encoded by *Br031692* and *Bra019972* which belonged to the Sucrose Transporter (SUT 4) subfamily interact with 10 of the total 15 BrCB5s. It was reported that the Arabidopsis SUT4 (AtSUT4) was a functional interactive protein to 5 members of the AtCB5 family which include a total of 7 members [21]. A previous study also showed that an Apple Sucrose Transporter, MdSUT1, interacted physically with an apple cytochrome b5 (MdCYB5) in vitro and vivo, which was verified by the yeast two-hybrid, immunocoprecipitation, and bimolecular fluorescence complementation assays [22].

Additionally, there are 2 predicted proteins that can interact with 9 and 7 members of BrCB5s, respectively. One is Bra006538, a NADH-cytochrome b5 reductase, and another one is a FAH 2 protein (Fatty Acid Hydroxylase 2) encoded by *Bra013479.* It was reported that NADH-Cyt b5 reductase has the ability to specifically reduce the content of CB5 in the presence of NADH [23]. It was also reported that AtFAH1 and AtFAH2 physically interact with AtCb5s from where they obtain electrons [24].

Moreover, 10, 6, and 20 predicted functional proteins interact with 3, 2, and 1 members of BrCB5s, respectively, and BrCB5e and m have no predicted functional proteins with a score ≥ 0.6 which was given by the STRING online tool.

### 2.5. Expression Patterns of *BrCB5s* in Various Tissues, in Response to Abiotic Stresses and Hormone Treatments

**Figure 3:**
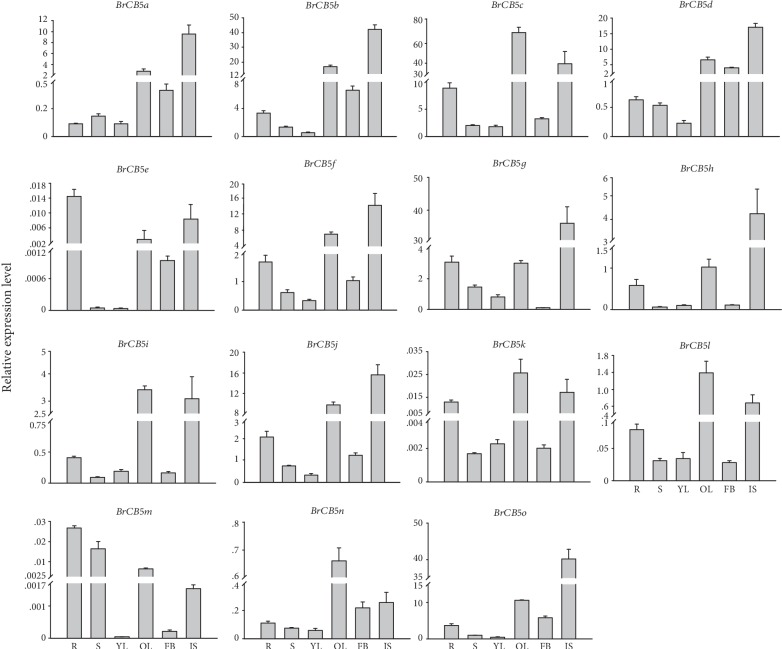
Expression analysis of the *BrCB5* genes in different tissues of Chinese cabbage. The surveyed tissues include root (R), stem (S), young leaf (YL), old leaf (OL), flower bud (FB), and immature silique (IS). The analysis was carried out by qRT-PCR. Expression levels of the *BrCB5* genes were normalized to those of *BrACT1*, and the 2^-*ΔΔ*Ct^ method was used to calculate the expression levels of target genes in different tissues.

To explore the functions of the *BrCB5s* in Chinese cabbage growth and development, mRNA expression analysis was performed in six tissues, including root (R), stem (S), young leaf (YL), old leaf (OL), flower bud (FB), and immature silique (IS). According to the results, all the 15 *BrCB5s* had a higher expression in the old leaves than in the young leaves; all the *BrCB5s* had a higher expression level in the immature silique than that in the stem; all the *BrCB5s* had a higher expression in the roots than in the stems, except *BrCB5a* and *BrCB5d*, which had a similar expression level in both tissues (Figure 3). In addition, eight of fifteen *BrCB5s* (*BrCB5a*, *BrCB5b*, *BrCB5d*, *BrCB5f*, *BrCB5g*, *BrCB5h*, and *BrCB5j*) were expressed mainly in immature siliques. There were five *BrCB5s* (*BrCB5c*, *BrCB5i*, *BrCB5k*, *BrCB5l*, and *BrCB5n*), and two *BrCB5s* (*BrCB5e* and *BrCB5m*) were expressed mainly in old leaves and in roots, respectively. Some paralogs showed a similar expression pattern in different tissues such as *BrCB5g/j* and *BrCB5d/b*, while some exhibited different expression tendencies in different tissues, for example, *BrCB5c/e*, *BrCB5h/i*, and *BrCB*5*k/m*. The varied expression patterns of the *BrCB5s* indicated that they might play different roles in the growth and development of those tissues.

**Figure 4:**
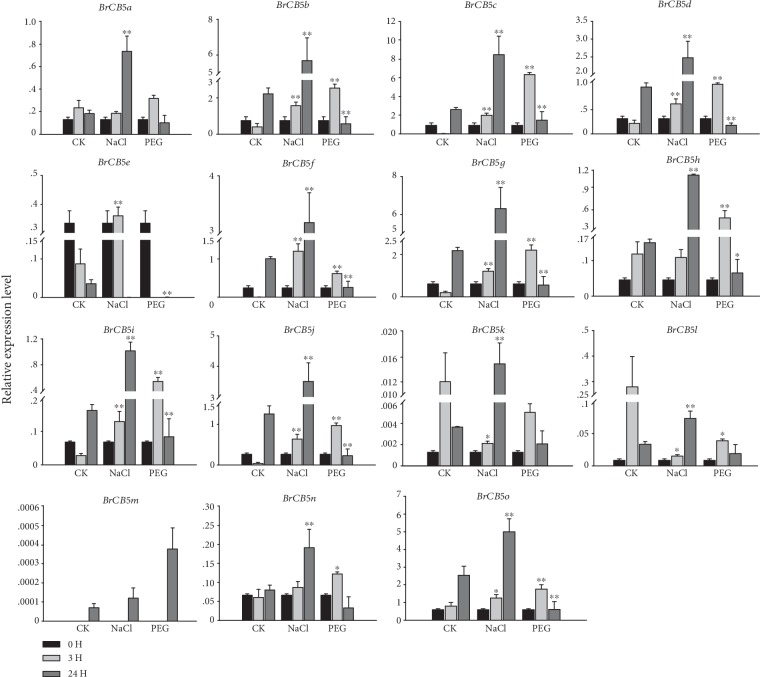
Expression analysis of the *BrCB5* genes under salt stresses. Three-week-old plants were treated with 20% (*w*/*v*) PEG_6000_ and 200 mM NaCl for 0, 3, and 24 h before the mature leaves were harvested. CK plants were treated with the same quantity water compared with salt. Expression of the *BrCB5* genes was normalized to those of *BrACT1* and shown relative to the expression of CK at 0 h. The 2^-*ΔΔ*Ct^ method was used to calculate the expression levels of target genes in different tissues. ∗ indicated that the expression level is significantly different from the value of the control (^∗^*p* < 0.05, ^∗∗^*p* < 0.01).

**Figure 5:**
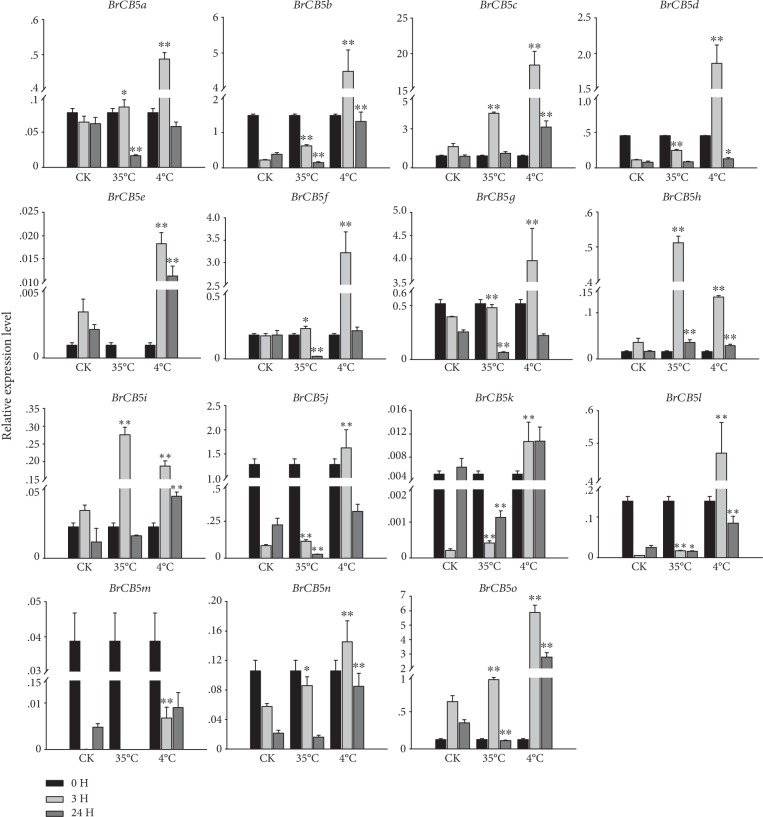
Expression analysis of the *BrCB5* genes under temperature stresses. Three-week-old plants were treated with 35°C and 4°C for 0, 3, and 24 h before the mature leaves were harvested. CK plants were treated with 22°C. Expression of the *BrCB5* genes was normalized to those of *BrActin* and shown relative to the expression of CK at 0 h. The 2^-*ΔΔ*Ct^ method was used to calculate the expression levels of target genes in different tissues. ∗ indicated that the expression level is significantly different from the value of the control (^∗^*p* < 0.05, ^∗∗^*p* < 0.01).

The responses of the *BrCB5s* to abiotic stresses, such as the common osmotic reagent (polyethylene glycol, PEG_6000_), salt (NaCl), heat (35°C), and cold (4°C) stress, were investigated to understand the potential roles of those *BrCB5* genes. The expression of these *BrCB5s* displayed different abundances under different stresses (Figures 4 and 5). For example, under the NaCl treatment, nine *BrCB5s* (*BrCB5b*, *BrCB5c*, *BrCB5d*, *BrCB5f*, *BrCB5g*, *BrCB5i*, *BrCB5j*, *BrCB5n*, and *BrCB5o*) were upregulated at 3 h and 24 h after the treatment, whereas three *BrCB5s* (*BrCB5a*, *BrCB5k*, and *BrCB5l*) were downregulated and upregulated at 3 h and 24 h after the treatment. *BrCB5*e was upregulated at 3 h while it was downregulated at 24 h after the treatment. The expression of *BrCB5h* was at a similar level with that of the CK group, while it was upregulated by six-fold compared with the control at 24 h. Under the PEG_6000_ stress, the expression pattern of the *BrCB5s* was basically the same, which was upregulated at 3 h and downregulated at 24 h after the treatment, respectively. *BrCB5k* had the same trend with most of the *BrCB5s* under PEG_6000_ stress, but there were no significance differences due to its low expression levels on the time points we selected. Specially, the expression of *BrCB5e* had a downward trend. In addition, *BrCB5m* expressed an extremely low level under both NaCl and PEG_6000_ treatments indicating that it might be not functional in either of the two signaling pathways.

During the 4°C treatment, nine *BrCB5s* (*BrCB5b*, *BrCB5c*, *BrCB5e*, *BrCB5i*, *BrCB5k*, *BrCB5l*, *BrCB5m*, *BrCB5n*, and *BrCB5o*) were upregulated at both 3 h and 24 h after the treatment compared with those in the CK group. At the same time, the proteins had the highest expression level at 3 h after the treatment, which was dramatically upregulated compared with that at 0 h and 24 h. For example, *BrCB5c*, *BrCB5d*, and *BrCB5f* were upregulated by 11, 9, and 16-fold at 3 h, respectively. All of the *BrCB5s* showed a significantly upward trend 3 hours after the 4°C treatment except *BrCB5m* with an opposite trend showing a significant decline of its expression level. In addition, *BrCB5k* had a sustained high level of expression from 3 h to 24 h after the low-temperature treatment. Under the 35°C treatment, there were three tendencies of the expression of *BrCB5s*. Firstly, seven *BrCB5s* (*BrCB5b*, *BrCB5d*, *BrCB5j*, *BrCB5k*, *BrCB5l*, *BrCB5m*, and *BrCB5n*) displayed a declined expression from 0 h to 24 h after the high-temperature treatment. Among these genes, *BrCB5b*, *BrCB5d*, *BrCB5j*, and *BrCB5l* had no obvious difference on the expression level between the experimental group and the CK group at the same time point after the treatment, while *BrCB5k* was downregulated by six-fold compared with that in the CK group at 24 h after the treatment. Secondly, four *BrCB5s* (*BrCB5c*, *BrCB5h*, *BrCB5i*, and *BrCB5o*) showed an increased expression level at 3 h but a decreased expression level at 24 h. Among these genes, *BrCB5h* and *BrCB5i* were upregulated by approximately 10-fold and 7-fold, respectively. The other three *BrCB5s* (*BrCB5a*, *BrCB5f*, and *BrCB5g*) only showed a decreased expression level at 24 h after the treatment, while the expression was stable between 0 h and 3 h after the treatment. *BrCB5e* and *BrCB5m* were expressed too low to analyze the significance under 35°C treatment. Considering the low expression level, it is probably that *BrCB5m* confers no specific function in Chinese cabbage under 35°C stress.

**Figure 6:**
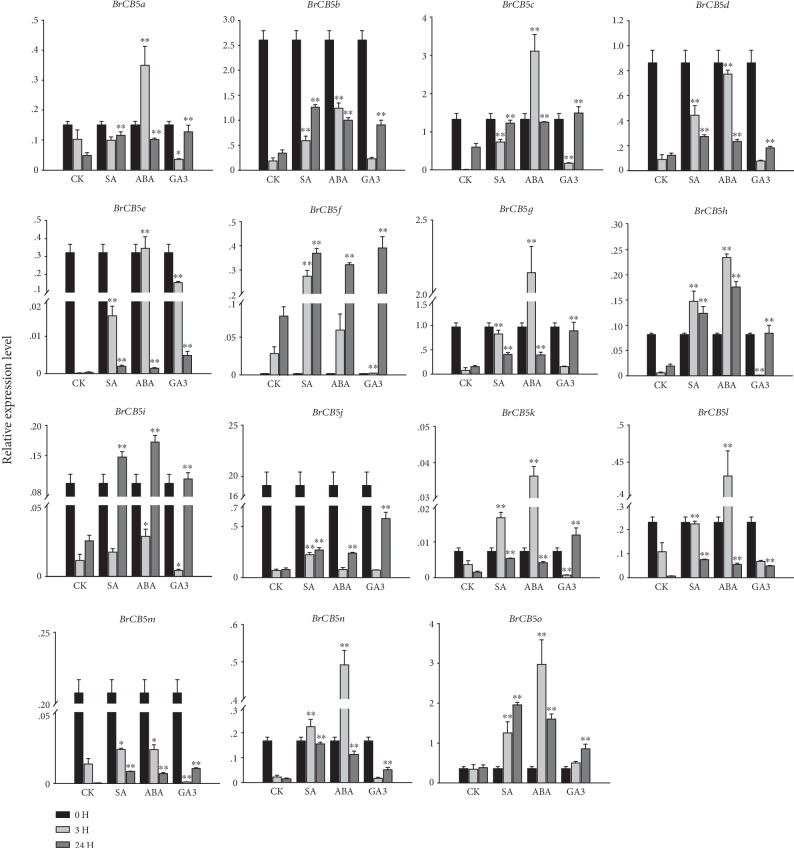
Expression analysis of the *BrCB5* genes under phytohormone treatment. Three-week-old plants were treated with 200 *μ*M GA3, 100 *μ*M ABA, and 200 *μ*M SA for 0, 3, and 24 h before the mature leaves were harvested. Expression of the *BrCB5* genes was normalized to those of *BrACT1* and shown relative to the expression of CK at 0 h. The 2^-*ΔΔ*Ct^ method was used to calculate the expression levels of target genes in different tissues. ∗ indicated that the expression level is significantly different from the value of the control (^∗^*p* < 0.05, ^∗∗^*p* < 0.01).

It is known that plant hormone plays crucial roles in plant growth and defense signaling. In order to explore the expression pattern of *BrCB5s* under plant hormone stress, Gibberellin A3 (GA3), Abscisic Acid (ABA), and Salicylic Acid (SA) were used to treat Chinese cabbage plants (Figure 6).

For the SA treatment, the expression levels of 13 *BrCB5s* were higher than that of the CK group at 3 h and 24 h, including *BrCB5b*, *BrCB5c*, *BrCB5e*, *BrCB5f*, *BrCB5g*, *BrCB5h*, *BrCB5i*, *BrCB5j*, *BrCB5k*, *BrCB5l*, *BrCB5m*, *BrCB5n*, and *BrCB5o*. *BrCB5a* showed no difference of expression at 3 h compared with that at 0 h, and *BrCB5d* was downregulated at 24 h after the SA treatment. Generally, the expression pattern of most *BrCB5s* showed a declining tendency from 0 h to 24 h after the treatment except *BrCB5f*, *BrCB5h*, *BrCB5i*, *BrCB5k*, *BrCB5n*, and *BrCB5o*. *BrCB5f* and *BrCB5o* had an upward trend in expression while the expression of the other genes increased at 3 h and then declined at 24 h.

Under the ABA treatment, compared with the CK group, the expression levels of most *BrCB5s* were upregulated, while the expression of *BrCB5f* and *BrCB5i* was almost the same at 3 h with that of the CK group. *BrCB5m* showed a specific expression pattern with a basically unchanged expression level compared with that in the CK group at both 3 h and 24 h. There were four genes (*BrCB5b*, *BrCB5d*, *BrCB5j*, and *BrCB5m*) which were downgraduated by about 3-fold, 9-fold, 100-fold, and 20-fold from 0 h to 24 h, respectively, while the expression level of *BrCB5f* increased by 6-fold. Nine genes showed the highest expression level at 3 h, including *BrCB5a*, *BrCB5c*, *BrCB5e*, *BrCB5g*, *BrCB5h*, *BrCB5k*, *BrCB5l*, *BrCB5n*, and *BrCB5o*. *BrCB5i* reached the highest expression level at 24 h after a 10-fold decline in expression at 3 h. Under the GA3 treatment, three *BrCB5s* (*BrCB5e*, *BrCB5g*, and *BrCB5o*) were upregulated, and one *BrCB5* gene (*BrCB5j*) maintained the same expression level at both 3 h and 24 h compared with that in the CK group. The other genes of the *BrCB5* family showed a fluctuated expression pattern. For example, *BrCB5f*, *BrCB5h*, *BrCB5i*, *BrCB5k*, *BrCB5m*, and *BrCB5n* were downregulated at 3 h, but upregulated at 24 h; *BrCB5d* maintained the same expression level with that in the CK group at 3 h, and then the expression decreased at 24 h. In the experimental group, under the GA3 treatment, the expression level of two genes (*BrCB5f* and *BrCB5o*) increased continuously and that of three genes (*BrCB5d*, *BrCB5e*, and *BrCB5l*) declined. The others had a significantly decreased expression level at 3 h, and then the expression increased at 24 h. For instance, the expression level of *BrCB5i* was declined by more than 20-fold at 3 h and then increased by almost 22-fold at 24 h.

Generally, the expression levels of *CB5s* were higher compared with that of the CK group at the same time point after treatment, respectively, but a conspicuously declined trend was found in the experimental group. For instance, *BrCB5i* and *BrCB5j* showed their lowest expression levels 3 hours after the four phytohormone treatments, while *BrCB5e* and *BrCB5l* had their lowest levels 24 hours after the treatments. *BrCB5m* was not as unique as it used to be, for *BrCB5b*, *BrCB5d*, and *BrCB5j* showed a similar downward trend under all these stresses.

## 3. Discussion

The *CB5* genes may play an important role in Chinese cabbage. However, there is little information about the function of the BrCB5s. It is unknown how the BrCB5s regulate growth and development and how they respond to a variety of biotic and abiotic stresses. To better and accurately explore the function of BrCB5s, we carried out bioinformatics analyses according to our previous studies [25, 26], which have been successfully applied to genome-wide analysis of GRF and VQ family genes in Chinese cabbage. Additionally, expression patterns of *BrCB5s* under some conventional abiotic and hormone treatments were carried out to explore the stress tolerance mechanisms of Chinese cabbage.

Genome duplication, which results in nonfunctionalization, subfunctionalization, and neofunctionalization, plays a vital role in expanding genome content and diversifying gene function [27–29]. Although the whole genome triplication (WGT) event was undergone by *Brassica rapa* [30] after its divergence from *Arabidopsis thaliana*, the *B. rapa* genome is approximately 4-fold larger than the *Arabidopsis* genome, and its gene number is twice than that of *Arabidopsis* [31, 32], suggesting that a large number of genes were lost during the genome duplication. In our study, we found 15 *BrCB5s* had a syntenic relationship with 7 *AtCB5s* (Table 1). Moreover, the WGT event had greatly expanded the gene family members of *B. rapa*. The phylogenetic and gene duplication analyses showed that the BrCB5s without tandem duplication contained two triplets, four duplicates, and one singleton. These results are similar with the *A. thaliana* genome, in which a large proportion of gene families are divided into low tandem and high segmental duplication class [33]. In our study, there were also some paralogs showing different expression patterns, for example, *BrCB5h*/*BrCB5i* and *BrCB5c*/*BrCB5e* in different tissues and *BrCB5a*/*BrCB5m* and *BrCB5b*/*BrCB5o* under various abiotic and hormone stresses, revealing that *BrCB5* paralogs might be maintained by subfunctionalization. Our results also confirmed the previously reported finding that duplicate genes can develop divergent patterns of gene expression for stably maintaining by subfunctionalization [34].

The expression patterns of *BrCB5s* in different tissues indicated that they might play important roles in the growth and development of specific tissues or organs. For instance, *BrCB5a*, *BrCB5b*, *BrCB5d*, *BrCB5h*, and *BrCB5o* had the highest expression level in immature siliques, suggesting that these genes are involved mainly in the growth and development of immature siliques in Chinese cabbage. Thus, we might improve Chinese cabbage seed harvest through regulating the expression of these genes.

Previous studies reported that CB5s interact with P450 enzymes either by supplying electrons or by modulating their activity via physical interaction independent of electron donation [11, 13]. It is known that P450s carry out essential enzymatic steps in the glucosinolate (GLS) biosynthetic pathway. *A. thaliana* produces GLS as their major class of specialized metabolites involved in plant defense [16, 35]. Additionally, it was confirmed that a CB5 protein, namely CB5C, can improve the efficiency of this pathway [15]. The GLS levels have been investigated using 2 mutants of the CB5C gene, and the results show that both mutants lead to subtle but distinct alterations in the levels of individual GLS. We explored the expression levels of some *BrGLS* genes under the NaCl treatment (Supplementary file 4. [Supplementary-material supplementary-material-1] and Supplementary file 6. [Supplementary-material supplementary-material-1]). The results showed that there existed some correlations between the two gene families under NaCl stress. Based on these results, GLS was used as an indicator to explore the role of CB5s in plants under stresses.

Salinity is considered the major abiotic stress affecting plant physiology and development [36, 37]. In our study, we found that, under the salt treatment, most of *BrCB5s* were upregulated at both time points we selected (Figure 4). One study showed that the GLS content is increased after 5 days under NaCl stress in *B. rapa* [38], which is consistent with our results. However, little is known about the expression of *CB5* under PEG_6000_ stress. We found that most *BrCB5s* were greatly upregulated at 3 h and then downregulated at 24 h upon the PEG_6000_ treatment. Water stress increases the glucosinolate accumulation in *Brassica* species—*Nasturtium officinale* L. [39], *Brassica oleracea* L. var. *capitata* [40], *Brassica oleracea* L. var. *italica* [41, 42], *Brassica napus* L. [43], *Brassica rapa* ssp. rapifera L. [44], and *Brassica carinata* L. [45]. It suggests that the response of *BrCB5s* to PEG_6000_ was an instantaneous and rapid process.

It is reported that elevated temperatures (21-34°C) can increase the glucosinolate levels in *Brassica rapa* and low-medium temperatures (15–27°C) can decrease the glucosinolate levels [46]. However, in our study, we found that the expression of almost all *BrCB5s* was downregulated at 35°C, while the expression reached the highest at 3 h under the 4°C treatment (Figure 5). The discrepancy has been observed by Justen et al. [47], and they refer these differences to different growth conditions and distinct genotypes.

It is known that ABA and SA play critical roles in mediating plant defense responses against pathogens and abiotic stresses [48, 49]. ABA is mainly related to plant defense against abiotic stresses. Environment factors such as drought, salinity, cold, heat stress, and wounding have been reported to trigger the increase of the ABA level [50, 51]. SA plays an important role in response to biotic stresses as evidenced by the fact that pathogen infection can lead to an elevated level of SA [52]. Not all the stress-response signaling pathways are specific, and plenty of evidences had been provided for the cross-talk of ABA, SA with GAs in regulating plant defense responses [52, 53]. GAs act throughout the whole plant life cycle to promote the growth of organs via enhancing cell division and elongation and to promote developmental phase transitions containing seed dormancy and germination, juvenile and adult growth phases, and vegetative and reproductive development [54, 55].We also examined the expression profiles of the *BrCB5s* in response to exogenous GA3 (Figure 6). In our study, we found that the majority of the *BrCB5s* were dramatically either up- or downregulated under the SA and ABA treatments and the expression of most *BrCB5s* was downregulated under the GA3 treatment.

*BrCB5s* can be used as electron donors in various oxidation/reduction reactions in cells. It has also been found that *BrCB5s* regulate the balance of active oxygen (ROS) in plants and are involved in response to a variety of biotic and abiotic stresses. Our results revealed that *BrCB5s* were more sensitive to NaCl and PEG_6000_ treatments than heat and cold stresses. However, for *BrVQs*, the response seems to be the opposite [26]. We also found that the expression patterns of *BrCB5s* under NaCl and PEG_6000_ treatments were significantly different. The expression levels of *BrCB5s* peaked in a few hours under the PEG_6000_ treatment and then started to decrease, while the expression levels of *BrCB5s* continued to increase at 24 h when treated by NaCl. For the phytohormone treatment, we found that the expression of *BrCB5s* responded to SA, ABA, and GA3 stresses with different tendencies, indicating that *BrCB5s* might function in more than one signaling pathways and even mediate the cross-link of different pathways.

Additionally, some of *BrCB5s* under certain stresses were expressed at low levels, such as *BrCB5e* and *BrCB5m* under high-temperature treatment; it is also probably that they are not functional in Chinese cabbage without or with stress.

## 4. Conclusions

We identified 15 members of the Chinese cabbage CB5 gene family, which were classified into seven subfamilies. The phylogenetic relationships of the CB5s among Chinese cabbage, Rice, and *Arabidopsis* suggested that *BrCB5s* were more closely related to *AtCB5s* than *OsCB5s*. Phylogenetic and duplication event analysis suggested that whole genome duplication might be the main contributor to the expansion of the *BrCB5s*. The number of CB5 genes in Chinese cabbage was more than twice than that of *AtCB5s*. When treated with various abiotic stresses and hormone stimuli, differential expression of *BrCB5s* was observed. These data indicate that *BrCB5s* play an important role in plant growth and development. *BrCB5s* might mediate the cross-link between abiotic stresses and hormone signaling. Our work provides a basis for a further understanding of the characteristics and functions of the CB5 family in Chinese cabbage.

## 5. Methods

### 5.1. Identification and Comparison of CB5 Gene Family Members in Chinese Cabbage

Bioinformatics methods were carried out according to our previous studies [25, 26], which have been successfully applied to genome-wide analysis of GRF and VQ family genes in Chinese cabbage. Briefly, the nucleotide and protein sequences of BrCB5s were identified based on the *B. rapa* line *Chiifu* genome sequence (http://brassicadb.org) [31]. The amino acid sequences were aligned by the software DNAMAN 6.0.40 (Lynnon Biosoft, Quebec, QC, Canada). Intron/exon structure analysis was performed by the Gene Structure display Server (GSDS) (http://gsds.cbi.pku.edu.cn/). The GC content was calculated by DNASTAR (Madison, WI, USA). The number of amino acids, molecular weight (MW), and theoretical isoelectric point (pI) were computed by the ProtParam tool (http://web.expasy.org/protparam/). Phylogenetic trees were constructed by the MEGA 5 software using the neighbor-joining method with 1000 bootstrap replicates.

The SSR markers were detected by the SSRIT software (http://archive.gramene.org/db/markers/ssrtool) with the parameters adjusted for identification of perfect di-, tri-, tetra-, penta-, and hexanucleotide motifs with a minimum of 6, 5, 4, and 4 repeats, respectively. The distribution of the conserved motifs and domains was detected by the MEME suite (http://meme-suite.org/tools/meme). The Arabidopsis and rice GRF protein sequences were downloaded from the Arabidopsis Information Resource (TAIR: http://www.arabidopsis.org/) and the Institute for Genomic Research Rice Genome Annotation project (TIGR: http://www.tigr.org/), respectively.

### 5.2. Plant Growth and Treatments

Chinese cabbage cultivar “Zaohuangbai” plants were used in our study. The seeds were germinated in a glass petri dish with clean water at 20 ± 2°C for 24 h; then the glass petri dish was placed in a 4°C fridge for 15 days. After the vernalization, the seedlings were transferred into pots with soil and grown in a greenhouse at 20 ± 2°C with a 16 h light/8 h dark photoperiod. Artificial pollination was carried out after 7 weeks. 15 days after fertilization, samples were collected from root (R), stem (S), old leaf (OL, rosette leaves), young leaves (YL, cauline young leaves), flower bud (FB), and immature silique (IS) in three biological replicates for analyzing the expression of BrCB5s in different tissues.

For salinity, osmotic and hormone treatments, the seeds were germinated in a glass petri dish with clean water at 20 ± 2°C for 24 h. After germination, seedlings were transferred into pots with growth medium and grown in a greenhouse at 20 ± 2°C with a photoperiod of 16 h light and 8 h dark. Three-week-old seedlings of similar size were selected for the abiotic and hormone treatments. The plants were irrigated with 200 mM NaCl or 20% (*w*/*v*) polyethylene glycol (PEG_6000_) while the CK group was watered with distilled water until the solution flowed out from the bottom of the pot. For temperature stresses, seedlings were transferred to incubators at 35°C and 4°C, respectively. For hormone treatments, the plant leaves were sprayed doubled-sided with 200 *μ*M Gibberellin A3 (GA3), 100 *μ*M Abscisic Acid (ABA), and 200 *μ*M Salicylic Acid (SA) solutions, respectively, while leaves of the CK group were sprayed with distilled water until drops began to fall from the leaves' surface. The leaves of the treated seedlings were harvested after 0, 3, and 24 h of the above abiotic and hormone treatments.

All materials were immediately frozen in liquid nitrogen and stored at –80°C until RNA isolation.

### 5.3. RT qPCR Expression Analysis of *BrCB5s*

Total RNA was extracted from each sample using TRIzol reagent (Invitrogen, Carlsbad, CA, USA) and treated with RNase-free DNase I (Takara, Dalian, China) for 45 min according to the manufacturer's protocol. First-strand cDNA was synthesized using PrimeScript 1^st^ Strand cDNA synthesis Kit (Takara). Quantitative real-time PCR (qRT-PCR) was carried out using a SYBR Green Master mix (Takara, Dalian, China) on an IQ5 Real-Time PCR Detection System (Bio-Rad, Hercules, CA, USA). The qRT-PCR primers designed for the *BrCB5s* and actin gene (*BrACT1*) are listed in Supplementary file 6. [Supplementary-material supplementary-material-1], and the *BrACT1* was proved to be successful in being the internal control in the research published by Lee et al. [56] in 2103. So, *BrACT1* was used as a constitutive expression control in the qRT-PCR experiments. The PCR cycling conditions comprised an initial polymerase activation step of 95°C for 1 min, followed by 40 cycles of 95°C for 10 s and 60°C for 30 s. After each PCR run, a dissociation curve was designed to confirm the specificity of the product and to avoid the production of primer dimers. Three replicates of each sample were conducted to calculate the average Ct values. The relative expression level was calculated by the comparative 2^-*ΔΔ*Ct^ method.

## Figures and Tables

**Table 1:** Syntenic *BrCB5* genes between Arabidopsis and Chinese cabbage.

tPCK Chr^a^	Block	Arabidopsis gene	Chinese cabbage gene		
			LF^b^	MF1^c^	MF2^c^
tPCK1	B	*AtCB5f* (AT1G26340)	*BrCB5n*	*—*	*—*
tPCK3	J	*AtCB5d* (AT2G32720)	*BrCB5j*	*BrCB5g*	*BrCB5f*
tPCK3	J	*AtCB5a* (AT2G46650)	*BrCB5i*	*BrCB5h*	*—*
tPCK5	wb	*AtCB5b* (AT5G53560)	*—*	*BrCB5e*	*BrCB5c*
tPCK5	R	*AtCB5g* (AT5G17770)	*BrCB5o*	*BrCB5d*	*BrCB5b*
tPCK7	wa	*AtCB5c* (AT5G48810)	*BrCB5k*	*—*	*BrCB5l*
tPCK7	D	*AtCB5e* (AT1G60660)	*—*	*BrCB5a*	*BrCB5m*

**Table 2:** Chinese cabbage *BrCB5* gene family. *BrCB5 a* to *o* were assigned according to their distribution in the genome. The GC content was calculated by DNASTAR (Madison, WI, USA). The number of amino acids, molecular weight (MW), and theoretical isoelectric point (pI) were computed by the ProtParam tool (http://web.expasy.org/protparam/). The rest of data were downloaded from the Brassica database (http://brassicadb.org).

Gene	Accession no.	Chr. (strand)	Start/stop codon	CDS (bp)	GC content (%)	Length^∗^ (aa)	MW^∗^ (kDa)	pl^∗^
*BrCB5a*	*Bra031489*	A01(-)	16850047/16850412	366	41.53	121	13.49	5.26
*BrCB5b*	*Bra023636*	A02(-)	3272221/3274222	756	42.28	251	27.89	8.27
*BrCB5c*	*Bra022660*	A02(-)	7173079/7173774	405	44.69	134	15.05	4.97
*BrCB5d*	*Bra006419*	A03(-)	3427963/3429928	849	44.41	282	31.37	8.23
*BrCB5e*	*Bra029062*	A03(-)	6128228/6129069	357	43.14	118	13.52	6.64
*BrCB5f*	*Bra022898*	A03(-)	7650181/7651235	405	41.98	134	15.10	5.49
*BrCB5g*	*Bra021809*	A04(-)	14736345/14737749	405	42.72	134	15.07	5.13
*BrCB5h*	*Bra039268*	A04(+)	18924993/18925595	405	42.96	134	15.14	5.92
*BrCB5i*	*Bra004518*	A05(-)	595853/596423	405	40.49	134	15.07	5.68
*BrCB5j*	*Bra005564*	A05(+)	6249749/6251132	405	42.22	134	15.07	5.12
*BrCB5k*	*Bra037461*	A06(-)	20209973/20210752	417	49.88	138	14.96	4.53
*BrCB5l*	*Bra036160*	A09(+)	2148955/2149735	423	43.03	140	15.06	4.73
*BrCB5m*	*Bra027144*	A09(+)	9193855/9195418	606	24.59	201	23.21	5.51
*BrCB5n*	*Bra024721*	A09(+)	24567294/24567999	408	46.57	135	15.25	4.56
*BrCB5o*	*Bra002104*	A10(+)	11488694/11490486	846	45.86	281	31.24	7.68

^∗^Length, WM, and pI refer to the translated BrCB5 proteins.

**Table 3:** Simple sequence repeats (SSRs) predicted in the *BrCB5s.*

Gene	Motif	No. of repeats	SSR start	SSR end	Length^∗^	Intron/exon
*BrCB5b*	gt	4	1591	1598	2002	Intron
*BrCB5d*	tcc	4	97	108	1966	Exon
*BrCB5d*	ttta	4	1117	1132	1966	Intron
*BrCB5d*	at	5	782	791	1966	Intron
*BrCB5e*	ga	4	166	173	842	Intron
*BrCB5g*	tc	4	695	702	1405	Intron
*BrCB5g*	ga	4	1204	1211	1405	Exon
*BrCB5j*	ga	4	1183	1190	1384	Exon
*BrCB5k*	tc	6	100	111	780	Intron
*BrCB5l*	ct	4	103	110	781	Intron
*BrCB5l*	ctt	4	768	779	781	Exon
*BrCB5m*	gag	5	12	26	1564	Exon
*BrCB5m*	gag	4	52	63	1564	Exon
*BrCB5m*	aga	5	1186	1200	1564	Exon
*BrCB5n*	tc	5	98	107	706	Intron
*BrCB5o*	ta	5	311	320	1793	Intron
*BrCB5o*	ta	8	681	696	1793	Intron

^∗^Length of the gene from the start codon to the stop codon in the genomic sequence. SSRs were identified using SSRIT (http://archive.gramene.org/db/markers/ssrtool).

**Table 4:** Predicted functional partners of BrCB5s using the STRING Database (https://string-db.org/), which is known of predicting protein-protein interactions. The scores and description of the predicted functional partners were also downloaded from the database. Here, we selected predicted interacting proteins with a score more than 0.6.

Predicted functional partners	BrCB5 proteins (score ≥ 0.6)													Description of predicted functional partners
	BrCB5a	BrCB5b	BrCB5c	BrCB5d	BrCB5f	BrCB5g	BrCB5h	BrCB5i	BrCB5j	BrCB5k	BrCB5l	BrCB5n	BrCB5o	
Bra031692	●		●		●	●	●	●	●	●	●	●		AT1G09960, SUT4 (Sucrose Transporter 4)
Bra019972	●		●		●	●	●	●	●	●	●	●		
Bra035720		●		●									●	AT4G32360, NADP adrenodoxin-like ferredoxin reductase
Bra026504		●		●									●	AT5G23300, PYRD (pyrimidine d); dihydroorotate dehydrogenase
Bra009669		●		●									●	
Bra022246		●		●									●	AT3G17810, dihydroorotate dehydrogenase family protein
Bra021275		●		●									●	
Bra001684		●		●									●	
Bra035130		●		●									●	AT1G79610, NHX6|sodium proton exchanger
Bra007824		●		●									●	AT5G50375, CPI1 (cyclopropyl isomerase)
Bra000625		●												
Bra015335		●												AT1G04620, coenzyme F420 hydrogenase family
Bra006538	●			●	●	●			●	●	●	●	●	AT5G20080, NADH-cytochrome b5 reductase
Bra002104				●										AT5G17770, NADH-cytochrome b5 reductase
Bra006419													●	
Bra013479		●			●	●			●	●	●	●		AT4G20870, FAH2 (Fatty Acid Hydroxylase 2)
Bra005775					●			●						AT5G03630, ATMDAR2; monodehydroascorbate reductase
Bra034777											●			AT3G12120, FAD2 (Fatty Acid Desaturase 2)
Bra035720											●			AT4G32360, NADP adrenodoxin-like ferredoxin reductase
Bra023065			●				●	●						AT3G16340, PDR1; ATPase
Bra017241			●				●	●						AT2G36380, PDR6; ATPase
Bra007769			●									●		AT2G26070, RTE1 (Reversion-to-Ethylene Sensitivity1)
Bra026434			●											AT4G26455, WIP1 (WPP-Domain Interacting Protein 1)
Bra013361			●											AT4G18910, NIP1, 2 (NOD26-Like Intrinsic Protein 1, 2)
Bra012567			●											
Bra038838												●		AT2G19080, metaxin-related
Bra038562												●		
Bra038877												●		AT3G51040, RTH (RTE1-Homolog)
Bra009383												●		AT5G09420, ATTOC64-V
Bra032473												●		AT1G05270, TraB family protein
Bra039818							●	●						AT2G14750, Adenylyl-sulfate kinase
Bra017872							●	●						
Bra013120							●	●						
Bra007769							●	●						AT2G26070, RTE1 (Reversion-to-Ethylene Sensitivity1)
Bra013376	●													AT4G19150, ankyrin repeat family protein
Bra023091	●													AT2G37020, sequence-specific DNA binding
Bra019738	●													AT1G12050, fumarylacetoacetase
Bra028859	●													AT5G02890, transferase family protein
Bra028858	●													
Bra009559	●													

## Data Availability

The data used to support the findings of this study are included within the article.

## Supplementary Materials

Supplementary 1 Supplementary file 1. Figure S1: locations of the BrCB5s on the Chinese cabbage chromosomes. The chromosome number is indicated at the top of each chromosome representation.

Supplementary 2 Supplementary file 2. Figure S2: conserved domains and motifs in BrCB5s. Note: (A) phylogenetic tree of Chinese cabbage, rice, and Arabidopsis CB5s. (B) Distribution of conserved motifs in Chinese cabbage, rice, and Arabidopsis BrCB5 proteins. (C) The sequence logos of predicted domains in the BrCB5 protein sequences downloaded from the MEME suite (http://meme-suite.org/tools/meme).

Supplementary 3 Supplementary file 3. Figure S3: multiple sequence alignment of the protein sequences of BrCB5s. Note: motifs 1, 2, and 3 were marked artificially according to the results of MEME, which were shown in supplementary Figure S1 (C). Different colors mean different similarities of amino acids on the same location of these protein sequences. The BrCB5 protein sequences were aligned using the DNAMAN software, in which red, blue colors indicated the similarity was above 75%, 50%, respectively.

Supplementary 4 Supplementary file 4. Figure S4: expression analysis of the BrGLS genes under NaCl stress. Note: the black, gray, and dark gray columns represent the expression levels of genes at 0, 3, and 24 h after NaCl treatment, respectively. ∗ indicated that the expression level is significantly different from the value of the control (^∗^*p* < 0.05, ^∗∗^*p* < 0.01).

Supplementary 5 Supplementary file 5. Table S1: the RT-qPCR primers designed for BrCB5s and BrACT1. The gene accession numbers shown in this table are the same with those shown in Table 2. The last line in this table was primers of BrACT1, which was used as a constitutive expression control in the RT-qPCR experiments.

Supplementary 6 Supplementary file 6. Table S2: the RT-qPCR primers designed for BrGLSs. The gene numbers, at ortholog, location, and function shown in this table were downloaded from the Brassica database (http://brassicadb.org/brad/index.php). The primers of BrACT1 used in this RT-qPCR experiment were the same as the primers in Supplementary file 5. [Supplementary-material supplementary-material-1].
