# Supplementary material for: Genome-Wide Identification and Analysis of the Cytochrome B5 Protein Family in Chinese Cabbage (Brassica rapa L. ssp. Pekinensis)
Source: Int J Genomics. 2019 Dec 2;2019:2102317. doi: 10.1155/2019/2102317 (PMC6913312; doi:10.1155/2019/2102317)
Supplement: Supplementary 1 — Supplementary file 1. Figure S1: locations of the BrCB5s on the Chinese cabbage chromosomes. The chromosome number is indicated at the top of each chromosome representation. [file 2102317.f1.docx]

A09

*BrCB5l*

*BrCB5m*

*BrCB5n*

A06

*BrCB5k*

A01

*BrCB5a*

A10

*BrCB5o*

A05

*BrCB5j*

*BrCB5i*

A04

*BrCB5h*

*BrCB5g*

A03

*BrCB5d*

*BrCB5e*

*BrCB5f*

A02

*BrCB5b*

*BrCB5c*

Supplementary file 1. Figure S1. Locations of the *BrCB5s* on the Chinese cabbage chromosomes. The chromosome number is indicated at the top of each chromosome representation.
