# Supplementary material for: Genome-Wide Identification and Analysis of the Cytochrome B5 Protein Family in Chinese Cabbage (Brassica rapa L. ssp. Pekinensis)
Source: Int J Genomics. 2019 Dec 2;2019:2102317. doi: 10.1155/2019/2102317 (PMC6913312; doi:10.1155/2019/2102317)
Supplement: Supplementary 2 — Supplementary file 2. Figure S2: conserved domains and motifs in BrCB5s. Note: (A) phylogenetic tree of Chinese cabbage, rice, and Arabidopsis CB5s. (B) Distribution of conserved motifs in Chinese cabbage, rice, and Arabidopsis BrCB5 proteins. (C) The sequence logos of predicted domains in the BrCB5 protein sequences downloaded from the MEME suite (http://meme-suite.org/tools/meme). [file 2102317.f2.docx]

Motif location

AtCB5d

BrCB5g

BrCB5j

BrCB5f

AtCB5c

BrCB5k

BrCB5l

OsCB5h

OsCB5n

OsCB5a

AtCB5a

BrCB5h

BrCB5i

OsCB5l

OsCB5b

BrCB5e

AtCB5b

BrCB5c

AtCB5f

BrCB5n

OsCB5e

OsCB5f

BrCB5m

AtCB5e

BrCB5a

OsCB5r

OsCB5j

OsCB5k

OsCB5i

OsCB5d

OsCB5c

OsCB5m

OsCB5g

OsCB5q

OsCB5p

OsCB5o

BrCB5d

BrCB5o

AtCB5g

BrCB5b


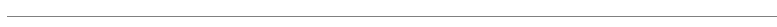

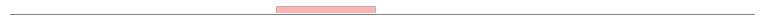

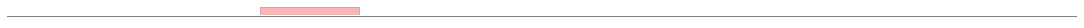

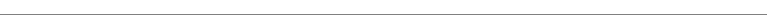

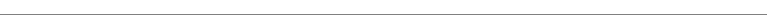

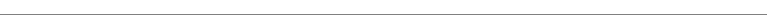

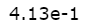

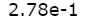

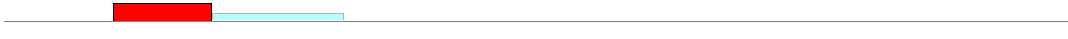

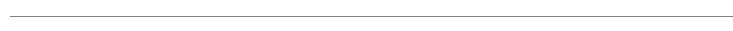

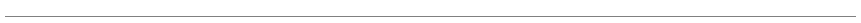

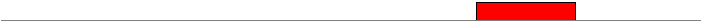

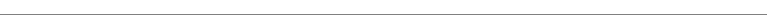

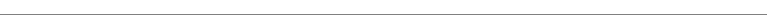

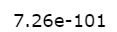

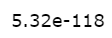

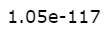

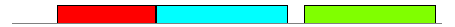

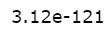

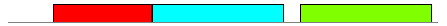

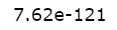

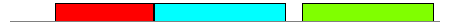

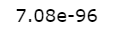

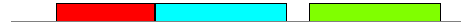

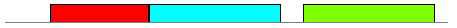

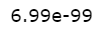

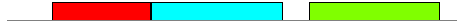

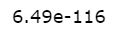

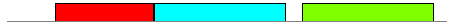

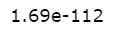

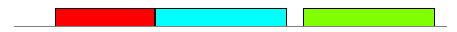

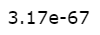

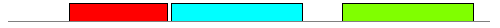

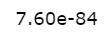

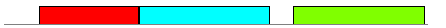

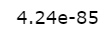

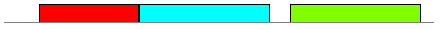

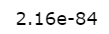

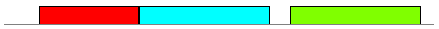

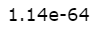

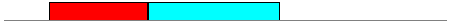

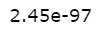

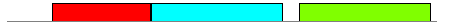

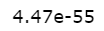

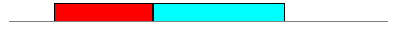

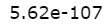

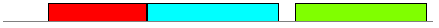

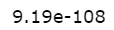

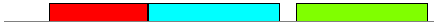

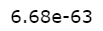

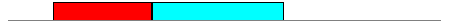

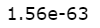

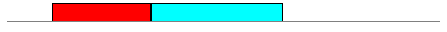

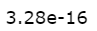

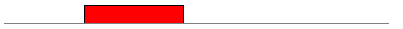

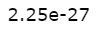

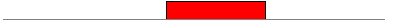

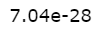

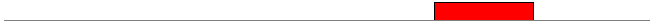

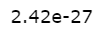

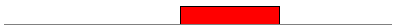

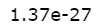

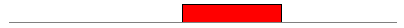

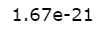

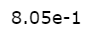

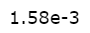

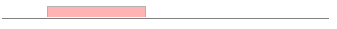

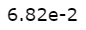

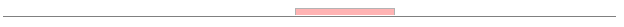

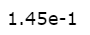

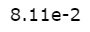

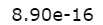

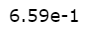

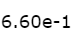

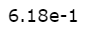

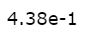

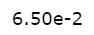

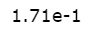

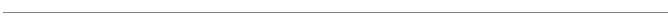

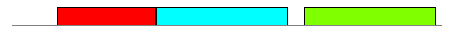


A

B


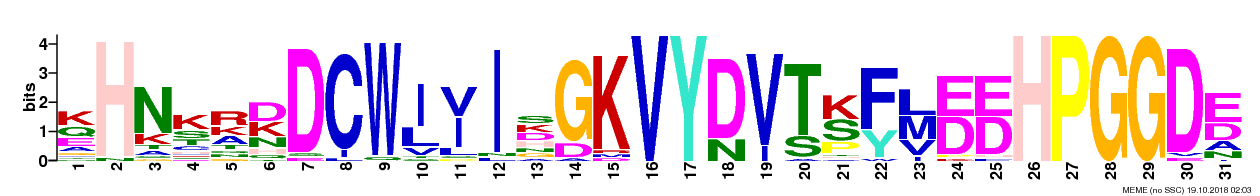

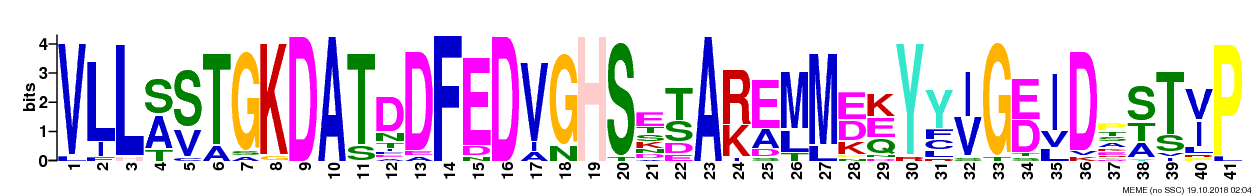

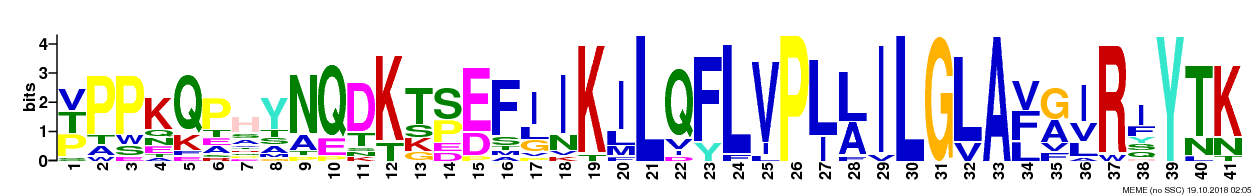


Motif1

Motif 2

Motif 3

C

*p-value*

Supplementary file 2. Figure S2. Conserved domains and motifs in BrCB5s.

(A) Phylogenetic tree of Chinese cabbage, rice, and Arabidopsis CB5s. The BrCB5 protein sequences of Chinese cabbage, rice and Arabidopsis were obtained from the BRAD (http://brassicadb.org/brad/), TIGR (http://www.tigr.org/) and TAIR (http://www.arabidopsis.org/), respectively. The phylogenetic tree was constructed with the MEGA 5 software using the neighbor-joining method with 1000 bootstrap replicates.

(B) Distribution of conserved motifs in Chinese cabbage, rice and Arabidopsis BrCB5 proteins. The distribution of the conserved motifs and domains were detected using the MEME suite (http://meme-suite.org/tools/meme), from where p-value and Motif location map were downloaded. The sequence logos of predicted domains in the BrCB5 protein sequences were shown in (C) as Motif 1, Motif 2 and Motif 3.
