# Supplementary material for: Genome-Wide Identification and Analysis of the Cytochrome B5 Protein Family in Chinese Cabbage (Brassica rapa L. ssp. Pekinensis)
Source: Int J Genomics. 2019 Dec 2;2019:2102317. doi: 10.1155/2019/2102317 (PMC6913312; doi:10.1155/2019/2102317)
Supplement: Supplementary 3 — Supplementary file 3. Figure S3: multiple sequence alignment of the protein sequences of BrCB5s. Note: motifs 1, 2, and 3 were marked artificially according to the results of MEME, which were shown in supplementary Figure S1 (C). Different colors mean different similarities of amino acids on the same location of these protein sequences. The BrCB5 protein sequences were aligned using the DNAMAN software, in which red, blue colors indicated the similarity was above 75%, 50%, respectively. [file 2102317.f3.docx]

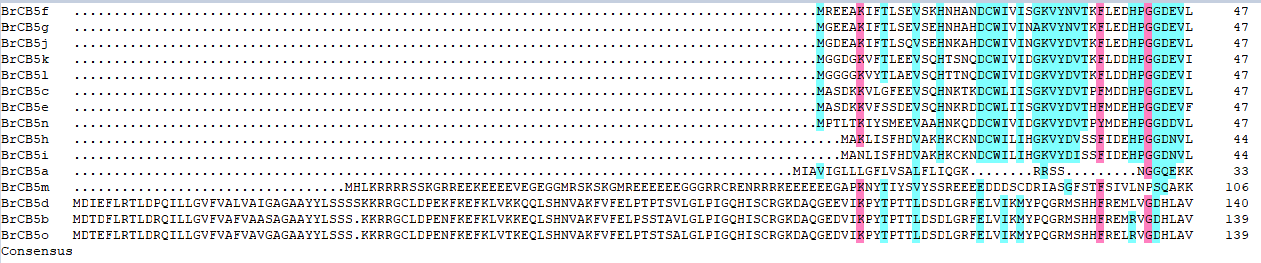


Motif 1

Motif 2

Motif 3


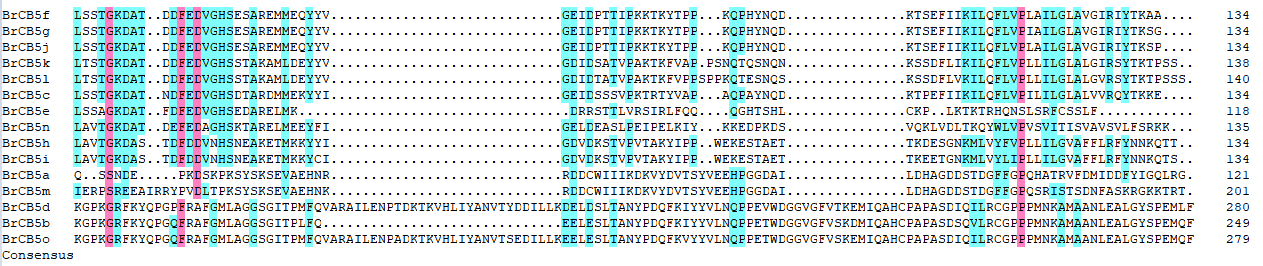


Supplementary file 3. Figure S3. Multiple sequence alignment of the protein sequences of BrCB5s. The sequences were aligned using the DNAMAN software, in which red, blue colors indicated the similarity was above 75%, 50%, respectively. Motif 1, 2, 3 were marked artificially according to the results of MEME, which were shown in supplementary figure S1 (C).
