# Supplementary material for: Genome-Wide Identification and Analysis of the Cytochrome B5 Protein Family in Chinese Cabbage (Brassica rapa L. ssp. Pekinensis)
Source: Int J Genomics. 2019 Dec 2;2019:2102317. doi: 10.1155/2019/2102317 (PMC6913312; doi:10.1155/2019/2102317)
Supplement: Supplementary 4 — Supplementary file 4. Figure S4: expression analysis of the BrGLS genes under NaCl stress. Note: the black, gray, and dark gray columns represent the expression levels of genes at 0, 3, and 24 h after NaCl treatment, respectively. ∗ indicated that the expression level is significantly different from the value of the control (∗p < 0.05, ∗∗p < 0.01). [file 2102317.f4.docx]

﹡﹡

*Bra031802*

CK

NaCl

0.00

.02

.08

.10

.12

﹡﹡

*Bra032734*

.25

.20

.15

.10

.05

Relative expression level

0.00

NaCl

CK

﹡﹡

*Bra015939*

.08

﹡﹡

*Bra012961*

CK

NaCl

0.00

.02

.04

.06

.08

.10

.06

.04

3H

24H

0H

.02

0.00

NaCl

CK

Supplementary file 4. Figure S4. Expression analysis of the *BrGLSs* under NaCl stress. Three-week-old plants were treated 200mM NaCl for 0, 3, 24 h before the mature leaves were harvested. CK plants were treated with distilled water. Expression of the *BrGLSs* were normalized to those of *BrACT1* and shown relative to the expression of CK at 0 h. The 2^-ΔΔCt^ method was used to calculate the expression levels of target genes in different tissue. * indicated that the expression level is significantly different from the value of the control (* p < 0.05, ** p < 0.01).
