# Supplementary material for: Genome-Wide Identification and Analysis of the Cytochrome B5 Protein Family in Chinese Cabbage (Brassica rapa L. ssp. Pekinensis)
Source: Int J Genomics. 2019 Dec 2;2019:2102317. doi: 10.1155/2019/2102317 (PMC6913312; doi:10.1155/2019/2102317)
Supplement: Supplementary 5 — Supplementary file 5. Table S1: the RT-qPCR primers designed for BrCB5s and BrACT1. The gene accession numbers shown in this table are the same with those shown in Table 2. The last line in this table was primers of BrACT1, which was used as a constitutive expression control in the RT-qPCR experiments. [file 2102317.f5.docx]

Supplementary file 5. Table S1. The RT-qPCR primers designed for *BrCB5s* and *BrACT1*. The gene accession numbers showed in this table were corresponded to that showed in Table 1. The last line in this table was primers of *BrACT1*, which was used as a constitutive expression control in the RT-qPCR experiments.

| **Gene Accession No.** | **F** | **R** |
| --- | --- | --- |
| Bra002104 | AGCCTCCGGAGACATGGGATG | AGCTTCAAGGTTTGCAGCCATGG |
| Bra004518 | CGGAAAGGACGCGTCGACTG | AGATTCTTTCTCCCACGGCGG |
| Bra005564 | ACTCCTCCTAAACAGCCTCACTACA | TCCCAACAGCCAAACCGAGAATG |
| Bra006419 | GCCTCCGGAAGTATGGGATGGT | GCTTCAAGGTTTGCAGCCATGG |
| Bra021809 | ACACACCTCCTAAACAGCCTCAC | ACGAATCCCAACTGCCAAACCG |
| Bra022660 | CCTGCTCAACCCGCTTACAACC | CGGACAACAAGAGCGAGACCC |
| Bra022898 | ACACACCTCCAAAACAGCCTCA | TGCTAAACCGAGAATGGCAAGGG |
| Bra023636 | AGCCTCCGGAGACATGGGATG | GCTTCAAGGTTTGCAGCCATGG |
| Bra024721 | TTTCATTGGCGAGCTTGACGAAG | GAGAACAAGACACTCACCGCAAC |
| Bra027144 | GGAGGAAGAAGAGGAGGAGGGAG | TCGCTTGGCTTGGATTGAGAACA |
| Bra029062 | AGGACAGAAGGAGTACTACATTGGTGA | GGTGTCTTGTCTTGGTTTTAAGTGGC |
| Bra031489 | CGCAGAGCACAACAGAAGAGACG | AGTCGCGTGTTGTGGTCCGA |
| Bra036160 | TCTCCTCCTAAGCAGACCGAGTC | GGACACCGAGAGCCAAGCCTA |
| Bra037461 | GGGAAAGACGCGACGGATGAC | GGGTCTGGTTTGAAGGAGGAGC |
| Bra039268 | TGTTCTCCTCGCCGTCACTGG | TGGAATATACTTCGCCGTCACCG |
| Bra022356（*BrACT1*） | CGTACTACCGGTATTGTGCT | GAGCTGGATTTGGAAGTCTC |
