# Supplementary material for: Genome-Wide Identification and Analysis of the Cytochrome B5 Protein Family in Chinese Cabbage (Brassica rapa L. ssp. Pekinensis)
Source: Int J Genomics. 2019 Dec 2;2019:2102317. doi: 10.1155/2019/2102317 (PMC6913312; doi:10.1155/2019/2102317)
Supplement: Supplementary 6 — Supplementary file 6. Table S2: the RT-qPCR primers designed for BrGLSs. The gene numbers, at ortholog, location, and function shown in this table were downloaded from the Brassica database (http://brassicadb.org/brad/index.php). The primers of BrACT1 used in this RT-qPCR experiment were the same as the primers in Supplementary file 5. . [file 2102317.f6.docx]

Supplementary file 6. Table S2. The RT-qPCR primers designed for *BrGLSs*. The gene numbers, At ortholog, location and function showed in this table were downloaded from Brassica Database (http://brassicadb.org/brad/index.php). The primers of *BrACT1* used in this RT-qPCR experiment was the same as primers in table S1.

| **Gene**  **(B. rapa)** | **At ortholog** | **Location** | **Function** | **Primer-F** | **Primer-R** |
| --- | --- | --- | --- | --- | --- |
| Bra012961 | AT5G61420 | A03:21326869…21328218 | Transcription factors | AGGCACCGCCTTGAACTGGT | TGCTGCGCCTAGTTCCGACA |
| Bra015939 | AT1G74080 | A07:23411313…23412768 | Transcription factors | GGACACCGTGTTGCAGAGC | GCCAACCTCCTTCACCGTGA |
| Bra032734 | AT4G13770 | A04:5460393…5462018 | Core structure formation | CATCGGGGTGGTGGCTCTAG | CCTGGCTAAGCTGGTGGAGG |
| Bra031802 | AT5G65940 | A02:26922952…26925642 | Co-substrate pathways | CTCGGTGCCAAGTTCTTCGC | GGACCATGGACGGAGACACC |
